# Separated at Birth? Microarray Analysis of Two Strikingly Similar *Yersinia* Species

**DOI:** 10.1002/cfg.195

**Published:** 2002-08

**Authors:** S. J. Hinchliffe, K. E. Isherwood, R. A. Stabler, P. C. F. Oyston, J. Hinds, R. W. Titbal, B. W. Wren

**Affiliations:** 1 The London School of Hygiene and Tropical Medicine Keppel Street London WC1E 7HT UK; 2 DSTL CBS Porton Down Salisbury, Wiltshire SP4 0JQ UK; 3 Department of Medical Microbiology St George's Hospital Medical School London SW17 0RE UK


**Comparative and Functional Genomics**
*Comp Funct Genom* 2002; **3**: 355–357.
Published online in Wiley InterScience (www.interscience.wiley.com). **DOI:** 10.1002/cfg.195

**Conference Review**

### Separated at birth? Microarray analysis of two strikingly similar *Yersinia* species

S. J. Hinchliffe,[1]* K. E. Isherwood,[2] R. A. Stabler,[3] P. C. F. Oyston,[2] J. Hinds,[3] R. W. Titball[2] and B. W. Wren[1]

[1] *The London School of Hygiene and Tropical Medicine, Keppel Street, London WC1E 7HT, UK*
[2] *DSTL, CBS Porton Down, Salisbury, Wiltshire SP4 0JQ, UK*
[3] *Department of Medical Microbiology, St George's Hospital Medical School, London SW17 0RE, UK*

**Correspondence to:*
*S. J. Hinchliffe, The London School of Hygiene and Tropical Medicine, Keppel Street, London WC1E 7HT, UK.*
*E-mail:*
*Stewart.Hinchliffe@lshtm.ac.uk*

**Keywords:** ***Yersinia*; microarray; genome**

Received: 31 May 2002
Accepted: 12 June 2002

The Black Death is possibly the most infamous pandemic in human history, which killed one-third of the European population and subsequently shaped Western civilization (reviewed in [5]). Epidemics occurred in relentless cycles up to the seventeenth century, until severe depopulation caused a gradual decline in cases. All this was caused by a single pathological agent, the Gram-negative bacterium, *Yersinia pestis*.

The Black Death was the second of three plague pandemics, and was thought to be caused by the *Y. pestis* biovar Medievalis. The first pandemic was the Justinian plague, thought to be caused by the *Y. pestis* biovar Antiqua, which resulted in devastation throughout the Middle East and Mediterranean basin during the sixth to eighth centuries. The final pandemic, caused by biovar Orientalis, began in 1855 in China, and killed millions of people during the nineteenth and twentieth centuries. Approximately 2000 cases are reported to the World Health Organization each year. *Y. pestis* is still endemic in some parts of the world, although public health measures and antibiotics have all but eliminated plague as a human disease from most developed countries.

It is curious that the closest relative to *Y. pestis*, *Yersinia pseudotuberculosis*, usually causes mild gastroenteritis in humans. Despite the fact that these species cause remarkably different diseases, they are very closely related at the genetic level (99% nucleotide identity for most shared genes), and data based on multiple locus sequence typing (MLST) analysis suggests that *Y. pestis* diverged from *Y. pseudotuberculosis* only 1500–20 000 years ago [1]. Essentially, *Y. pseudotuberculosis*, a mild gut pathogen, has evolved into *Y. pestis*, which has devastated the human race, in an eye-blink of evolutionary time. The major genetic difference between the two species appears to be the acquisition of two plasmids by *Y. pestis*. Pathogenic *Y. pseudotuberculosis* retains a single plasmid (pCD1), whereas *Y. pestis* also retains two extra plasmids (pMT1 and pPCP1). It is therefore widely accepted that *Y. pestis* was once a simple enteropathogen, and acquiring these two plasmids, and other chromosomally located genes, allowed it

Copyright © 2002 John Wiley & Sons, Ltd.

to change its environmental niches and its lifestyle. This has been comprehensively demonstrated by the mutation of the gene *ymt* on pMT1, which encodes for a phospholipase D. This mutant is severely attenuated in both murine and flea models [2,3].

The complete genome sequence of *Y. pestis* CO92 (biovar Orientalis) was published in 2001 [4], and revealed a multitude of genes likely to be relevant to its pathogenesis and evolution. However, the genome sequence has revealed more questions than answers. Over 30% of coding sequences are of unknown function, and many more appear to have been acquired from other bacteria and even viruses. Very little is known as to how any of these genes feature in the ability of the organism to adapt to life in a mammalian host or in the flea. The unusually large number of insertion sequences present in *Y. pestis* encompasses approximately 3.7% of the genome, 10 times more than in *Y. pseudotuberculosis.* This high frequency of gene acquisition has led to a highly fluid genome, with large inversions occurring between IS elements [4]. A large number of pseudogenes are also present in the *Y. pestis* genome, disrupted by IS elements, or by other mutations. These pseudogenes are thought to have been required for an enteropathogenic lifestyle, but are now redundant.

In order to begin to make sense out of the vast amount of information generated by the sequencing of the CO92 genome, an analysis of global gene regulation and genome stability was required. Utilizing microarray techniques, we could analyse genomic DNA from a variety of *Y. pestis* and *Y. pseudotuberculosis* strains in order to give insights into the evolution of the two species since their divergence, and to determine just how stable the *Y. pestis* genome is. We could also begin to compare how these two species have adapted to different environments and how they regulate their gene expression in response to various stimuli.

Using the published CO92 genome sequence, a *Y. pestis* gene-specific microarray was designed by the amplification of all coding sequences, using in-house primer design software in collaboration with the Bacterial Microarray Group at St George's Hospital Medical School, London. The primers were designed so that products from the polymerase chain reaction (PCR) would allow maximum hybridization for each individual gene, with minimum cross-reactivity with any other DNA sequence in the genome.

Initial tests of the microarray with Cy3-labelled CO92 genomic DNA showed good hybridization to all spots. Subsequent hybridizations with Cy5-labelled genomic DNA from both *Y. pestis* and *Y. pseudotuberculosis* have also shown good hybridization, with low background. Comparative software used to analyse these arrays allows us to make reliable conclusions about the genes represented on the array despite any variation in the hybridization between spots (Imagene, BioDiscovery; Genespring, Silicon Genetics). The presence or absence of individual genes in a genomic DNA sample is easily distinguished, allowing conclusions to be drawn as to the evolution of the two species from a common ancestor. As with all genomic DNA-based microarray experiments, the presence of the gene in the genome does not mean that the gene is functional. The known pseudogenes of CO92 hybridize equally well to the array as the known functional genes. An example of this is *yapB*, a putative autotransporter protein and a pseudogene of CO92. According to the microarray data, this gene is present in the genome of the *Y. pseudotuberculosis* strain YPIII pIB1. PCR analysis demonstrates that only the terminal portion of this gene is present in the *Y. pseudotuberculosis* YPIII pIB1 genome, and this happens to be the region which is presented on the microarray.

The initial experiments, comparing genomic DNA from nine different *Y. pestis* strains to the CO92 control strain, highlighted the extreme fluidity and apparent instability of the *Y. pestis* genome and plasmids. *Y. pestis* strains were plated onto Congo Red agar, and single pigmented colonies were picked and grown up overnight in BAB broth. Genomic DNA was then purified and labelled with Cy5 dCTP before hybridization to the microarray. During the *in vitro* growth in BAB, six of the 15 strains appeared to lose the high pathogenicity island which encodes the pigmentation locus, along with the yersiniabactin gene cluster and other important genes. This indicates that this pathogenicity island is extremely unstable *in vitro* for most strains of *Y. pestis*, and is only maintained *in vivo* by its essential role in pathogenicity. Many of the strains have also lost one or more plasmids. These strains are all currently being passaged through a murine infection model before being rehybridized to the array. This is hoped to

Copyright © 2002 John Wiley & Sons, Ltd. 

enrich for bacteria that retain important virulence determinants.

Analysis of the *Y. pseudotuberculosis* strains by microarray has shown that they are more stable than the *Y. pestis* strains, with all 10 strains maintaining the pigmentation locus. This may be due to the absence of the neighbouring stretch of DNA in the high pathogenicity island which contains the yersiniabactin gene cluster. The acquisition of this stretch of DNA by *Y. pestis* has probably destabilized this portion of the genome by allowing recombination between IS elements.

Initial experiments have all been based around the variations in genomic DNA between strains of *Y. pestis* and *Y. pseudotuberculosis*. Future experiments aim to include the third pathogenic Yersinia species, *Yersinia enterocolitica*. This will enable us to study the evolution of the pathogenic Yersiniae and to determine the fundamental genetic changes in each species as they have adapted to new environmental niches.

However, genomic comparisons can only tell us so much about these organisms. Much more can be learnt using the microarray by studying the actual gene expression of the organism, and by comparing how these gene expression profiles differ between the pathogenic Yersiniae. Growth of these organisms in conditions to simulate various aspects of enteropathogenic and plague lifestyles will allow us to investigate the regulation of important genes and just how much of a leap it was for *Y. pestis* to adapt from being an enteropathogen to become a blood-borne pathogen with an insect vector.

The determination of the relative expression of genes under these particular conditions will allow the grouping of genes of unknown function with other known genes, which will allow predictions to be made as to their true function. Relatively simple experiments, such as the differences in gene expression in *Yersinia* grown at 28 °C when compared with growth at 37 °C, will ascertain genes important for survival in the flea and those important for survival in a mammalian host. Other potential comparisons include growth in limiting iron, magnesium or calcium, or at low pH.

Various regulatory mutants of the Yersiniae, such as PhoP-defective mutants, have also been generated by insertion of antibiotic resistance cassettes into specific genes. Comparison of these mutants with the wild-type, when grown under the appropriate conditions, will provide data on their exact roles in gene regulation.

Other *Yersinia* genome sequencing projects under way at present are *Y. pseudotuberculosis* (strain IP 32953; Lawrence Livermore National Laboratory, CA), a second *Y. pestis* genome sequencing project (strain KIM5 P12, biovar Medievalis; University of Wisconsin), and *Y. enterocolitica* (strain 8081; Sanger Institute, UK). Direct comparisons of the genome sequences of the three pathogenic Yersiniae will allow the development of a pan-species *Yersinia* microarray and lead to invaluable insights into how the enteropathogens are adapted to their lifestyle, and how *Y. pestis* was separated at birth to develop a new and completely different lifestyle.

### Acknowledgements

We acknowledge financial support for this project from DSTL and The Wellcome Trust. We also acknowledge BμG@S (the Bacterial Microarray Group at St George's) and The Wellcome Trust for funding their work; Keith Vass from the University of Glasgow; and Mike Prentice from St. Bartholomew's Hospital for his advice and expertise in all things *Yersinia*.

### References

1. Achtman M, Zurth K, Morelli G, Torrea G, Guiyoule A, Carniel E. 1999. *Yersinia pestis*, the cause of plague, is a recently emerged clone of *Yersinia pseudotuberculosis*. *Proc Natl Acad Sci USA* **96**(24): 14 043–14 048.
2. Hinnebusch J, Cherepanov P, Du Y, *et al.* 2000. Murine toxin of *Yersinia pestis* shows phospholipase D activity but is not required for virulence in mice. *Int J Med Microbiol* **290**: 483–487.
3. Hinnebusch BJ, Rudolph AE, Cherepanov P, Dixon JE, Schwan TG, Forsberg A. 2002. Role of *Yersinia* murine toxin in survival of *Yersinia pestis* in the midgut of the flea vector. *Science* **296**(5568): 733–735.
4. Parkhill J, Wren BW, Thomson NR, *et al.* 2001. Genome sequence of *Yersinia pestis*, the causative agent of plague. *Nature* **413**: 523–527.
5. Perry RD, Fetherston JD. 1997. Yersinia pestis — etiologic agent of plague. *Clin Micro Rev* **10**(1): 35–66.

Copyright © 2002 John Wiley & Sons, Ltd. 
